# Supplementary material for: Transcriptome Analysis Reveals Brassinolide Signaling Pathway Control of Foxtail Millet Seedling Starch and Sucrose Metabolism under Freezing Stress, with Implications for Growth and Development
Source: Int J Mol Sci. 2023 Jul 18;24(14):11590. doi: 10.3390/ijms241411590 (PMC10380969; doi:10.3390/ijms241411590)
Supplement: Supplementary file 1 [file ijms-24-11590-s001.zip › Figures S1-S4.pdf]

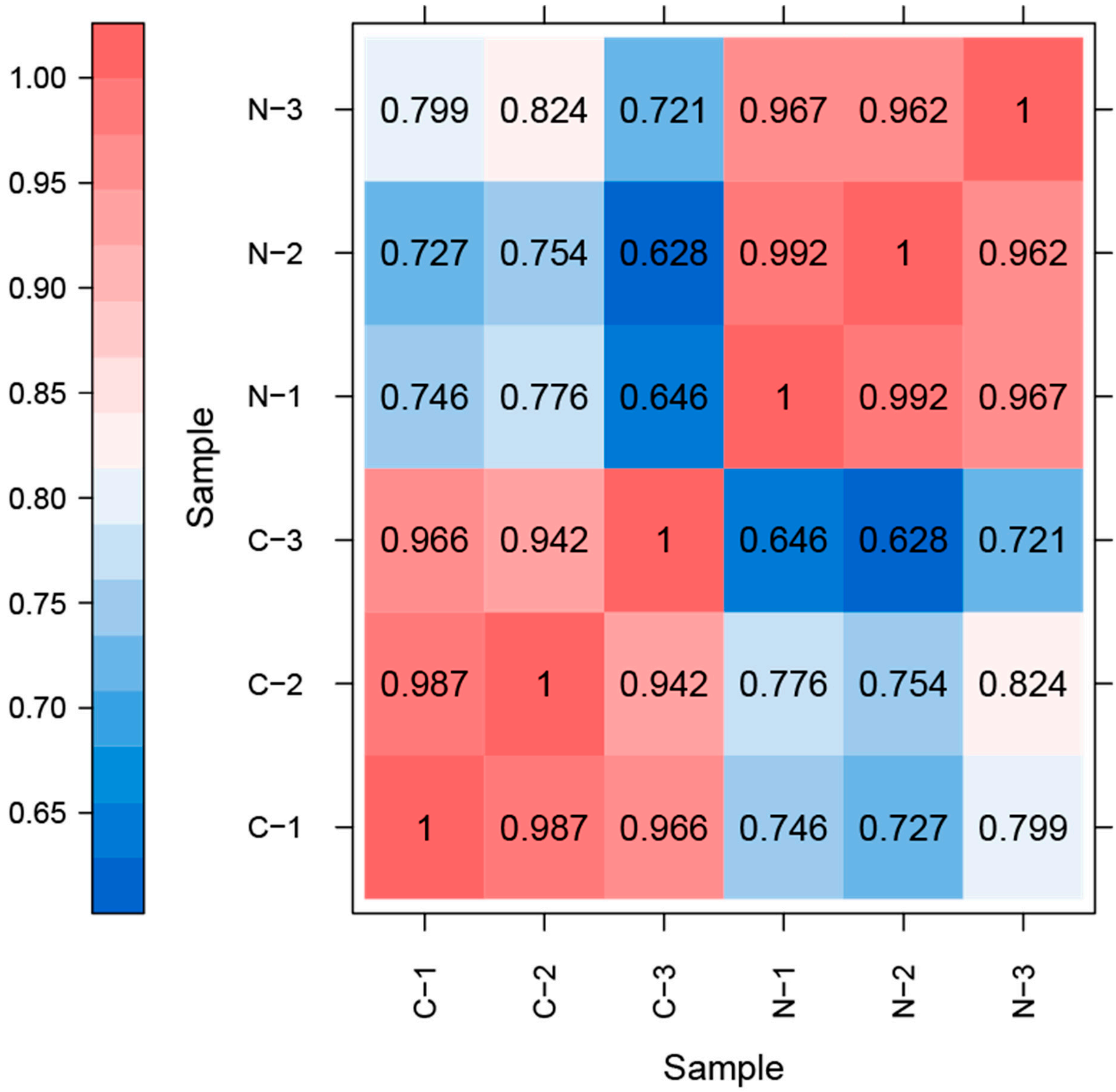

Figure S1. Correlations value of each repetition.

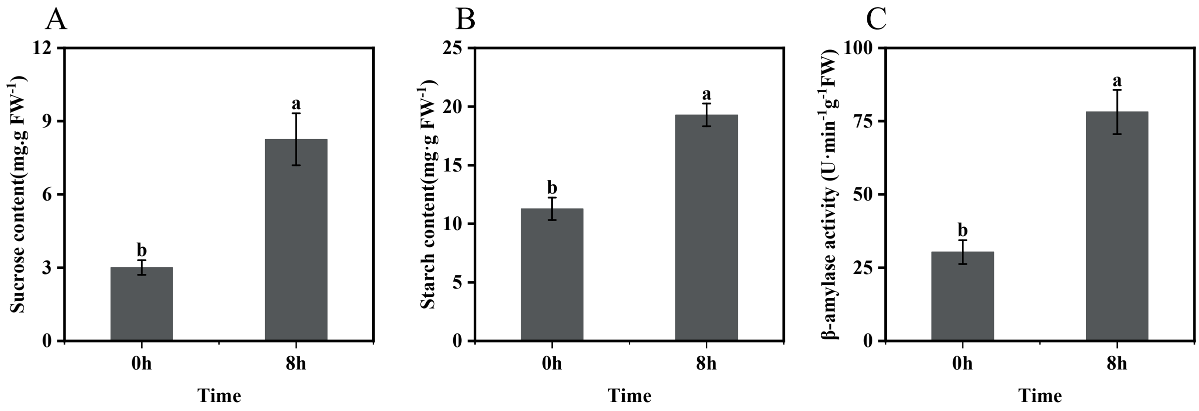

Figure S2. Sucrose content (**A**), Starch content (**B**) and β-amylase activity (**C**) of foxtail millet under −4 °C freezing stress.

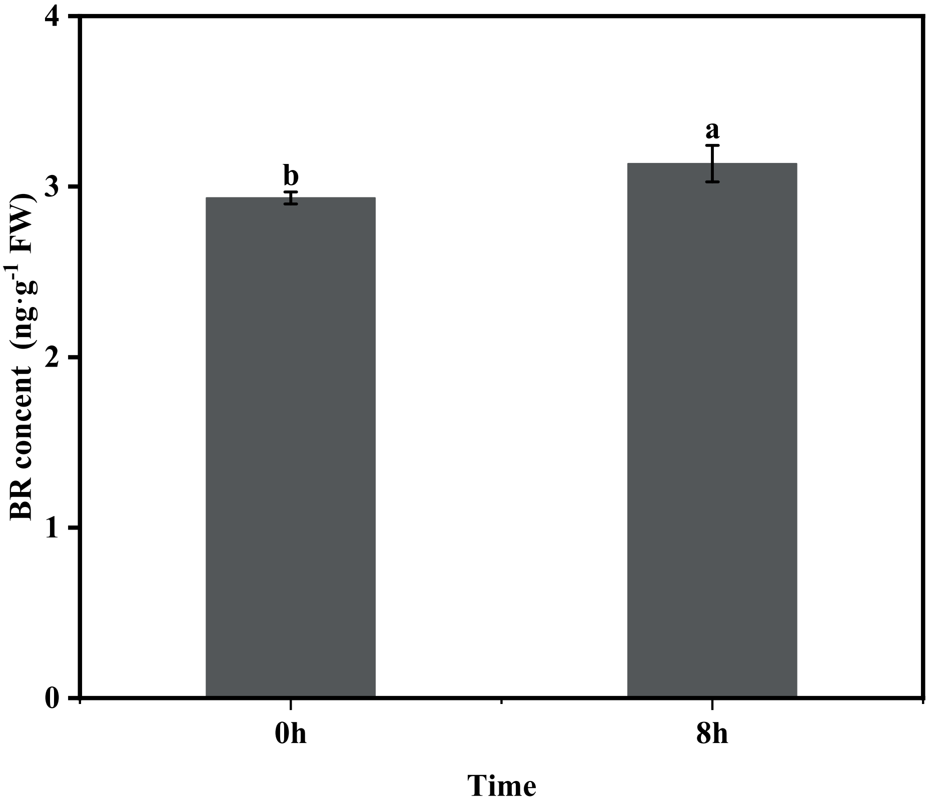

Figure S3. BR concent of foxtail millet under −4 °C freezing stress.

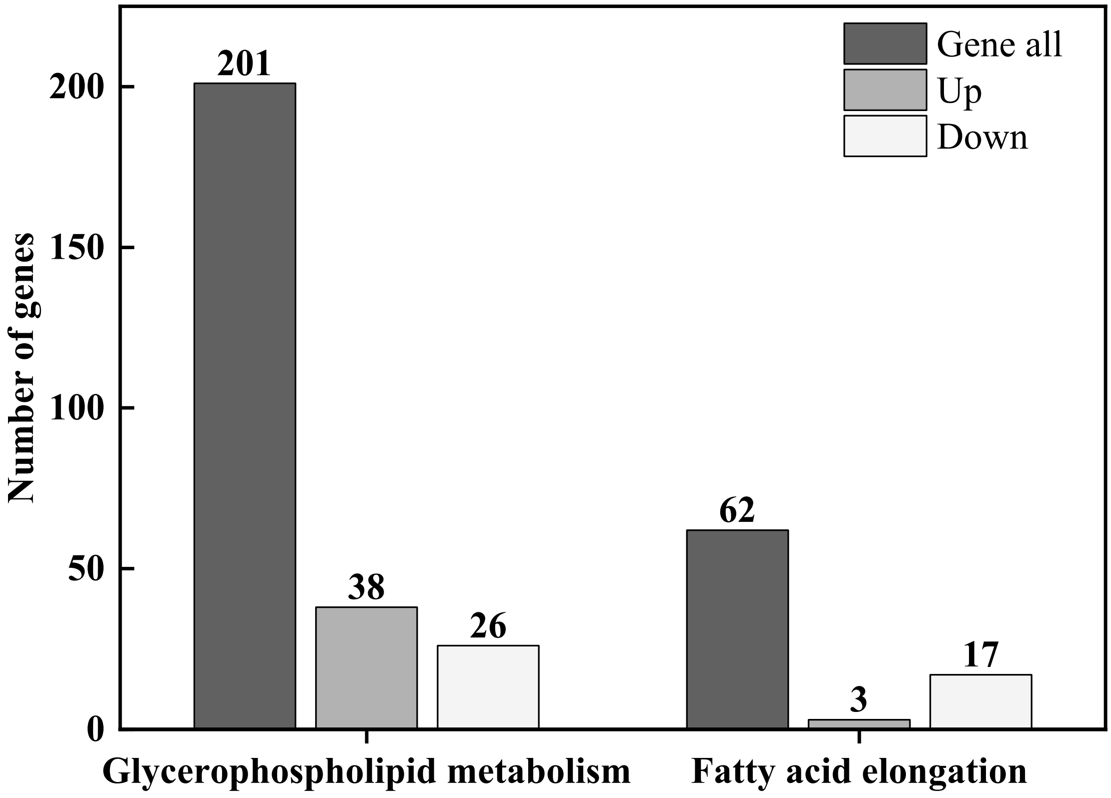

Figure S4. "fatty acid elongation (ko00564)"' and "glycerophospholipid metabolism (ko00062)" Statistical map of differential genes.
