# Supplementary material for: Transcriptome Analysis Reveals Brassinolide Signaling Pathway Control of Foxtail Millet Seedling Starch and Sucrose Metabolism under Freezing Stress, with Implications for Growth and Development
Source: Int J Mol Sci. 2023 Jul 18;24(14):11590. doi: 10.3390/ijms241411590 (PMC10380969; doi:10.3390/ijms241411590)
Supplement: Supplementary file 1 [file ijms-24-11590-s001.zip › Table S1.pdf]

Supplementary Table S1

Table S1. Primers for qRT-PCR

Gene ID	Gene Name	Forward Primer (5'-3')	Reverse Primer (5'-3')
Seita.1G027000	AP2/ERF	GCCGTTCTCTTTCCACGAC	TCGACGAACCCGAAGAGGTC
Seita.1G031300	PXMP2	GCAAGTGACATCTTCTCCCTGGAAC	AAGGCATGTACTGGTGGTTGATCC
Seita.1G154200	WRKY	GCCTGCCGCCTCCGATTATTAC	CACCGTGCTGCACTTGTAGTACC
Seita.1G236000	grxC	GGCGAATTACAAGGCTGTTGAACTG	GCCACCGATATGTTGCCCTTTAATG
Seita.1G257300	AP2/ERF	GCAAGTTCGCCGCAGAGATCC	GGACGAGGAGGACGACGAAGG
Seita.1G347300	TPS	CATGTTCGAGAGCATCGTGTG	CATCTTGATCACGTCCACGGT
Seita.3G221300	SCRK	GCTCAAGATCGACTCCCTTGT	GCTCAAGATCGACTCCCTTGT
Seita.6G032500	BRI1	TGCTCGTGGGCTTGCTTATCTTC	GTTCCTGCTACTCCAGTGCTCAC
Seita.6G181600	AMY	CGGGTCGACACAGAAGATGT	TCGTAGAAGATGCAGGGGAC
Seita.7G045300	bHLH	GCGGAGCGGACGAAGAAGATG	GGTGATGCTGGCGGTGATGAC
Seita.7G240400	BAK1	CGGATCTCAAGGTCAGCAACAAGG	GGATGACTGCGTGGGCGTTTC
Seita.8G202000	SOD2	CAAGACCGCTGCCCTATGATGATC	TCGAAGTGGTTCTGACTCCTCTGG
Seita.9G317500	BAM	TGGAGGAGATGAGCAGCAACCC	TGTAGTCGGCGTAGACCTGGATG
Seita.9G203800	grxD	CTGGTTGGTGGTTCCGACATAGTG	GAACTGAAGCCGCAACGAGGAG
Seita.9G544600	BAM	CAGGAAGAACGGGCTCAAGGTG	GAAGTCGGCGTAGCACTGGATG
SiActin	SETIT_026509mg	CGCATATGTGGCTCTTGACT	GGGCACCTAAATCTCTCTGC
